# Response to the Letter to the Editor

**DOI:** 10.1042/CS20260820

**Published:** 2026-05-22

**Authors:** Fouad A. Zouein, George W. Booz

**Affiliations:** 1 American University of Beirut, Beirut, Lebanon; 2 The University of Mississippi Medical Center, Department of Pharmacology and Toxicology, Jackson, Mississippi, U.S.A.

We thank the authors of the Letter to the Editor (DOI: 10.1042/CS20260449) for bringing this fascinating area of research to our attention. The studies cited provide evidence that lipid droplets and mitochondria physically interact. How the interplay between these two organelles is regulated, and the impact that this delivery system has on mitochondrial dynamics is an exciting area of investigation. More research needs to be done, but we agree that the knowledge gained may translate into new therapeutic possibilities.

## Open Access

Open access for this article was enabled by the participation of the University of Mississippi in an all-inclusive Read & Publish agreement with Portland Press and the Biochemical Society.

